# 2902. Dalbavancin Use in Bone and Joint Infections

**DOI:** 10.1093/ofid/ofad500.173

**Published:** 2023-11-27

**Authors:** Srivani Sanikommu, Liam P Alderson, Brett Bailey, Jeffrey Stambough, Benjamin Stronach, Simon Mears, Ryan K Dare

**Affiliations:** UAMS, little rock, AR; UAMS, little rock, AR; UAMS, little rock, AR; UAMS, little rock, AR; UAMS, little rock, AR; UAMS, little rock, AR; University of Arkansas for Medical Sciences, College of Medicine, Little Rock, Arkansas

## Abstract

**Background:**

Dalbavancin (DAL) is currently FDA-approved to treat acute bacterial skin and soft tissue infections. Use of DAL for off-label indications have been reported in difficult-to-treat infections, including osteomyelitis and prosthetic joint infections. Once weekly DAL dosing is an attractive alternative to daily intravenous (IV) infusions decreasing risks of prolonged home IV therapy. However, effectiveness for treatment of bone and joint infections is unknown

**Figure:**
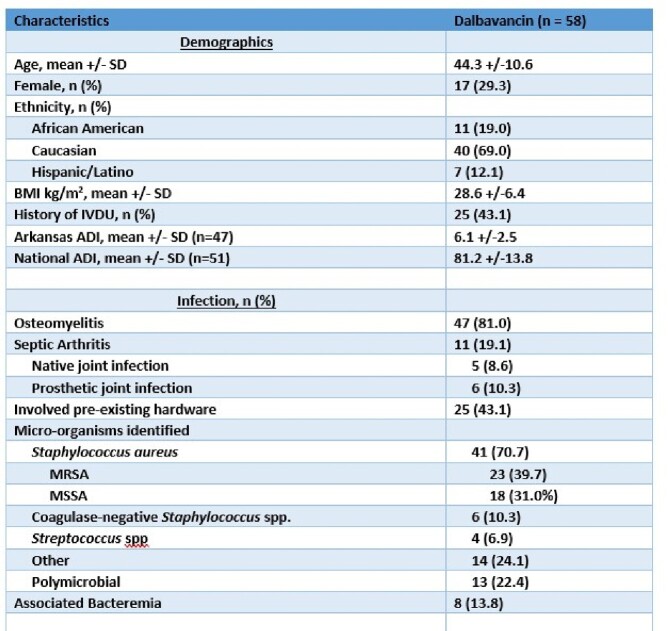

**Figure:**
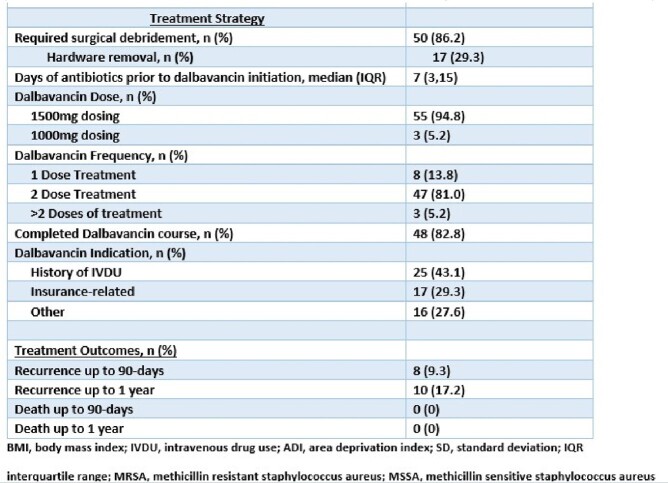

**Methods:**

This single-center, retrospective, observational study included adult patients who received at least one dose of DAL from 09/2019-03/2023 for bone and joint infections. Patient demographics including state and national area of deprivation index (ADI) data, underlying infection, dalbavancin indication, surgical and medical treatments, and clinical outcomes were obtained. Risk factor analyses were performed for 1-year infection recurrence using Stata 15.0 statistical software.

**Results:**

Fifty-eight patients were treated with DAL with a mean age was 44.3 years, with males comprising 71% with a mean BMI of 28.6. This vulnerable patient population had an Arkansas ADI decile of 6.1 and a national ADI percentile of 81.2. Osteomyelitis (81%), prosthetic joint infections (PJIs) (10.3%,), and native joint infections (8.6%) were included. Twenty-five (43.1%) patients had infected hardware involved. The majority (86.2%) of patients underwent surgical debridement and 17 (29%) required hardware removal, leaving 8 (13.8%) with retained hardware. Staphylococcus aureus was the most common organism identified with 39.7% and 31.0% MRSA and MSSA respectively. The most common DAL indication was IV drug use (43%). Recurrence of infection occurred in 8 patients (13.7%) within 90 days and 10 patients (17.2%) at one year. Hardware retention was noted to be a significant risk factor for recurrence (p=0.024), and hardware removal decreased risk of recurrence (p= 0.026). There was no recurrence in PJIs and there were no deaths noted.

**Conclusion:**

Our evidence suggests that DAL is an effective treatment option for bone and joint infections with debridement and hardware removal. Hardware retention increased risk for 1-year recurrence. Further evaluation with prospective, randomized controlled trials is needed.

**Disclosures:**

**All Authors**: No reported disclosures

